# Chromosomal instability of circulating tumor DNA reflect therapeutic responses in advanced gastric cancer

**DOI:** 10.1038/s41419-019-1907-4

**Published:** 2019-09-20

**Authors:** Zuhua Chen, Cheng Zhang, Mengqi Zhang, Beifang Li, Yunyun Niu, Limeng Chen, Jing Yang, Sijia Lu, Jing Gao, Lin Shen

**Affiliations:** 1 Key Laboratory of Carcinogenesis and Translational Research (Ministry of Education/Beijing), Department of Gastrointestinal Oncology, Peking University Cancer Hospital and Institute, Beijing, China; 2 Department of Clinical Research, Yikon Genomics Co. Ltd., Shanghai, China

**Keywords:** Predictive markers, Gastric cancer

## Abstract

Gastric cancer is characterized by chromosomal instability. In this study, we investigated chromosomal instability quantified by copy number instability (CNI) score of circulating tumor DNA (ctDNA) during the drug treatment in advanced gastric cancer (AGC). A total of 55 pretherapeutic plasmas from 55 AGC patients and 75 plasmas during drug treatment of 26 AGC patients were collected. Plasma ctDNA was extracted and assessed by whole-genome sequencing (WGS) for somatic copy number alteration (SCNA), and according to which we calculated the CNI scores. We next assessed the correlations between chromosomal instability and therapeutic response. The cutoff value of chromosomal instability was defined as the mean + SD of the CNI scores (56.60) in cfDNA of plasmas from 100 healthy people. For 55 enrolled cases, chromosomal instability was observed in 27 (49%) prior to drug treatment, whose response rate (59%, 16/27) was higher than in 28 patients with stable chromosomes (32%, 9/28, *P* = 0.043). We also observed that CNI scores fluctuated during treatment in 26 patients. Specifically, the CNI scores in 93% (14/15) of patients sensitive to drug treatment reduced to the level of chromosomal stability and the CNI scores in 52% (13/25) of patients resistant to treatment elevated again. For ctDNA with developed resistance, the SCNA patterns were identical to those before treatment, whereas the CNI scores were lower than the pretherapeutic scores. We found that chromosomal instability based on ctDNA could predict and monitor therapeutic response in gastric cancer, although validation in a larger cohort will be necessary.

## Introduction

Gastric cancer (GC) is featured by the high heterogeneity on anatomical, molecular, and cellular levels, which greatly impeded the current therapeutic development for advanced GC (AGC)^1,2^. Individualized chemotherapeutic or targeted therapy guided by predictive and monitoring markers has been progressed desirably into the era of precision medicine, yet the therapeutic targets for GC remains limited^3^. Although several landmark studies have highlighted the molecular subtypes of GC on DNA, RNA, and protein levels based on large-cohort tumor tissues, the application of such markers in clinical practice remains to be a big challenge^4–6^. Although predictive and resistance markers are equally important for systemic antitumor therapy, most research efforts to date have been focused on exploring the efficacy of predictive markers, while only a few clear markers of resistance have been found^7,8^. Meanwhile, due to the specific features of GC, the current Response Evaluation Criteria in Solid Tumors (RECIST) fail to evaluate drug resistance in a precisely and timely manner^9^.

Chromosomal instability, often indicated as large or small somatic gains and losses on chromosomal level, has been widely accepted as a hallmark of cancer^10^. A large amount drugs exert their antitumor activities through disrupting chromosomal stability^11^. Moreover, chromosomal instability has been reported to drive the clonal evolution of intratumoral heterogeneity, and confer the intrinsic and acquired resistance^11,12^. Hence, dynamically deciphering the landscape of chromosomal stability during treatment is of great importance in help understanding the therapeutic effect as well as cancer evolution. Nevertheless, the sequential acquisition of tumor tissues is clinically impractical, and new approach to achieve surveillance for patients’ response is urgently demanded.

With the rapid progress of high-throughput sequencing and liquid biopsy techniques, circulating tumor DNA (ctDNA) presented in plasma have become the alternative surrogate to tissues^13^. Genome-wide profiling of copy number instability (CNI) in ctDNA provides a suitable method to quantify chromosomal instability and predict therapeutic response in several cancers^14,15^. However, the significance and dynamic changes of chromosomal instability during the treatment of AGC have not been reported. Our previous research indicated that the *HER2* copy number detected by plasma ctDNA could serve as a response biomarker of trastuzumab treatment^16^. In the present study, we attempted to elucidate the chromosomal instability based on ctDNA during chemotherapy or targeted therapy by whole-genome sequencing and investigate their significance in AGC.

## Results

### The clinicopathological characteristics of 26 patients

A total of 55 patients were enrolled with a median age of 58 years (range: 29–80 years). Totally, 43 patients (78%) were male and 12 (22%) were female. Most of the patients were diagnosed with HER2-positive (55%, 30/55), poorly differentiated (51%, 28/55), and intestinal (69%, 38/55) AGC. All of the patients received at least two cycles of drug treatment with 30 patients receiving targeted therapy (pyrotinib, RC48, trastuzumab, pertuzumab, and fuquintinib) alone or in combination with chemotherapy and 25 patients undergoing chemotherapy alone. The detailed clinicopathological characteristics of 55 patients are shown in Table 1 and Table S1.

**Table 1:** The clinicopathological characteristics of patients (*N* = 55)

Characteristics	Number (%)
*Sex*	
Male	43 (78.2)
Female	12 (21.8)
*Age*	
<65	41 (74.5)
≥65	14 (25.5)
*Tumor location*	
EGJ	21 (38.2)
Non-EGJ	34 (61.8)
*Differentiation*	
High	4 (7.3)
Middle	23 (41.8)
Low	28 (50.9)
*Lauren classification*	
Intestinal type	38 (69.1)
Diffuse type	10 (18.2)
Mixed type	7 (12.7)
*HER2 status*	
Positive	30 (54.5)
Negative	25 (45.5)
*Targeted treatment*	
Yes	30 (54.5)
No	25 (45.5)

### SCNA patterns in individuals reflected tumor heterogeneity

**Fig. 1:**
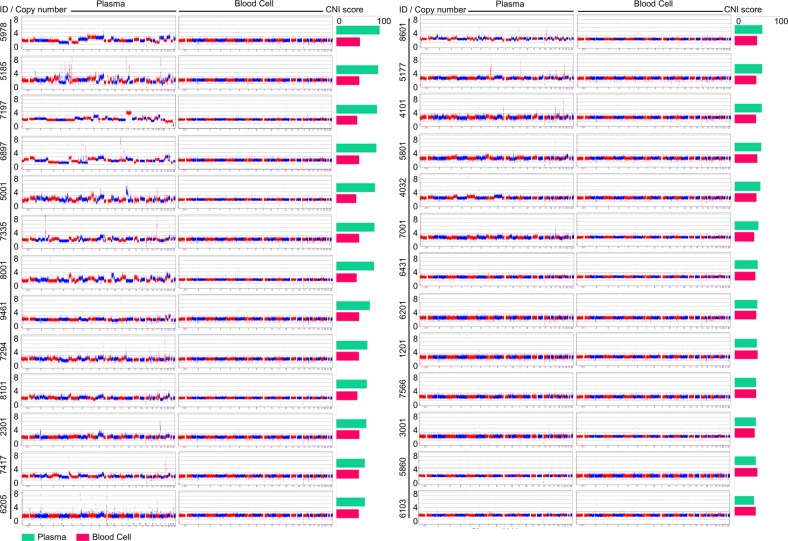
SCNV patterns of ctDNA at baseline among 26 patients. Genome-wide patterns of somatic copy number variation (SCNV) based on ctDNA of baseline plasma and corresponding blood cell samples from 26 patients were analyzed and plotted. The *x*-axis and *y*-axis represent the loci of 22 chromosomes and corresponding copy numbers, respectively. CNI score copy number instability score

Genome-wide patterns of SCNA detected in ctDNA derived from baseline plasma and paired blood samples showed high tumor heterogeneity among patients (Fig. 1 and Fig. S1). According to the profiling of segmented copy numbers, large-scale copy number gain or loss was observed on chromosomes 5, 7, 8, 13, 17, 18, 19, and 20 in ctDNAs of plasmas 5001, 7335, 5185, 8001, 5978, and 7294. Among the tumor-related genes, the top amplified genes included *ERBB2* (17q21), *MYC* (8q24), *GNAS* (20q13), *EGFR* (7p11), *ZNF217* (20q13), *CCNE1*(19q12), *NCOA3* (20q13), and *CDK6* (7q21), and the most frequently deleted genes were *CDKN2A* (9p21), *CDKN2B* (9p21), *KIT* (4q12), *SMAD4* (18q21), and *FGFR1* (8p12). Most of these genes encoded receptor tyrosine kinases and cell cycle-related proteins, which was consistent with the previously published studies on GC^4,17^.

### CNI scores of ctDNA in all patients

We calculated the CNI scores in ctDNA from 130 plasmas from 55 patients and cfDNA from 100 plasmas from 100 healthy people (Table S2). The cutoff value of the chromosomal instability was defined as the mean + SD of the CNI scores (56.60) in 100 plasmas from healthy people. Chromosomal instability with diverse patterns was observed in 27 of 55 patients (49%) prior to drug treatment.

**Fig. 2:**
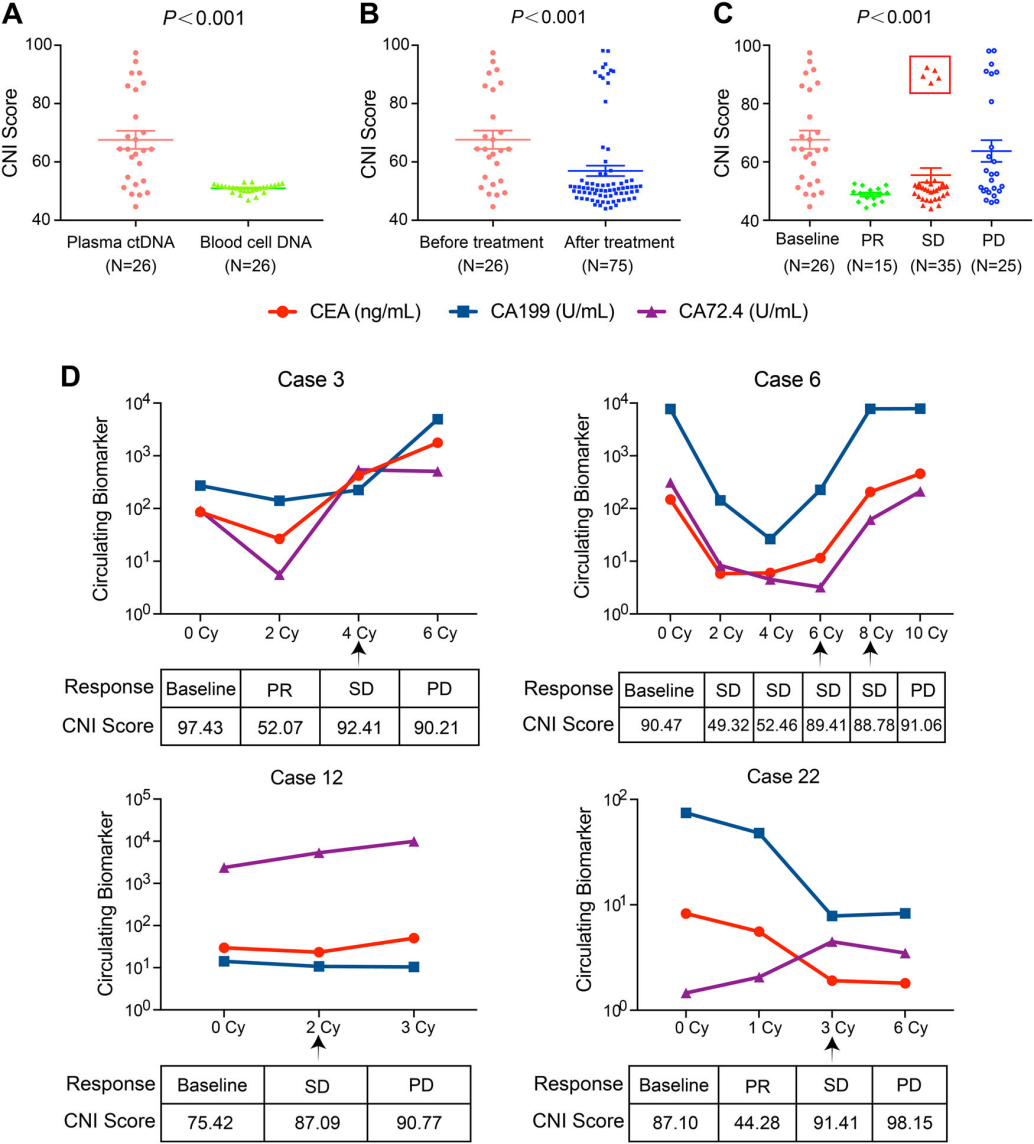
The CNI scores of ctDNA in 26 patients with dynamic plasma samples. **a** The CNI scores of ctDNA in plasmas and paired blood cells from 26 AGC patients. **b** The CNI scores of ctDNA from plasma samples before and after treatment in 26 AGC patients. **c** The CNI scores of ctDNA from plasma under different clinical responses. CNI score copy number instability score, PR partial response, SD stable disease, PD progressive disease. All data are presented as mean ± SD. *P* < 0.001 according to *t* test or one-way ANOVA. **d** The dynamic changes of CNI scores and tumor biomarkers during the administration of treatments in four patients

Among 26 patients with paired blood cells and dynamic plasma samples, the CNI scores of ctDNA derived from baseline plasmas were significantly higher than those from paired blood cells (67.62 ± 15.99 vs. 49.88 ± 2.47, *P* *<* 0.001) (Fig. 2a). The CNI scores after drug treatment were significantly lower than they were before treatment (56.94 ± 15.50 vs. 67.62 ± 15.99, *P* *<* 0.001) (Fig. 2b). Moreover, CNI scores fluctuated during therapy. 93% (14/15) of post-therapeutic ctDNA at the time of partial response (PR) had the lowest scores (48.85 ± 2.54), while 52% (13/25) of ctDNA with developed resistance to therapy retained elevated scores (63.75 ± 18.65). For ctDNA with developed resistance, SCNA patterns were identical to those before treatment, but the instability scores were lower than the pretherapeutic level (63.75 ± 18.65 vs. 67.62 ± 15.99) (Fig. 2c).

Five ctDNA from four patients at the timepoint of stable disease (SD) showed relatively high CNI scores (cases 3, 6, 12, and 22), which was analyzed individually (Fig. 2d). For case 3 and case 6, although the clinical response was determined as SD by CT after four or six cycles’ treatment respectively, the tumor biomarkers (CEA, CA199, and CA72.4) were increased, which suggested a trend toward disease progression. For case 12 and case 22, the increase of tumor biomarkers at the time of SD was not significant, and only CA72.4 increased slightly.

### Chromosomal instability of ctDNA prior to treatment could predict the therapeutic response

**Fig. 3:**
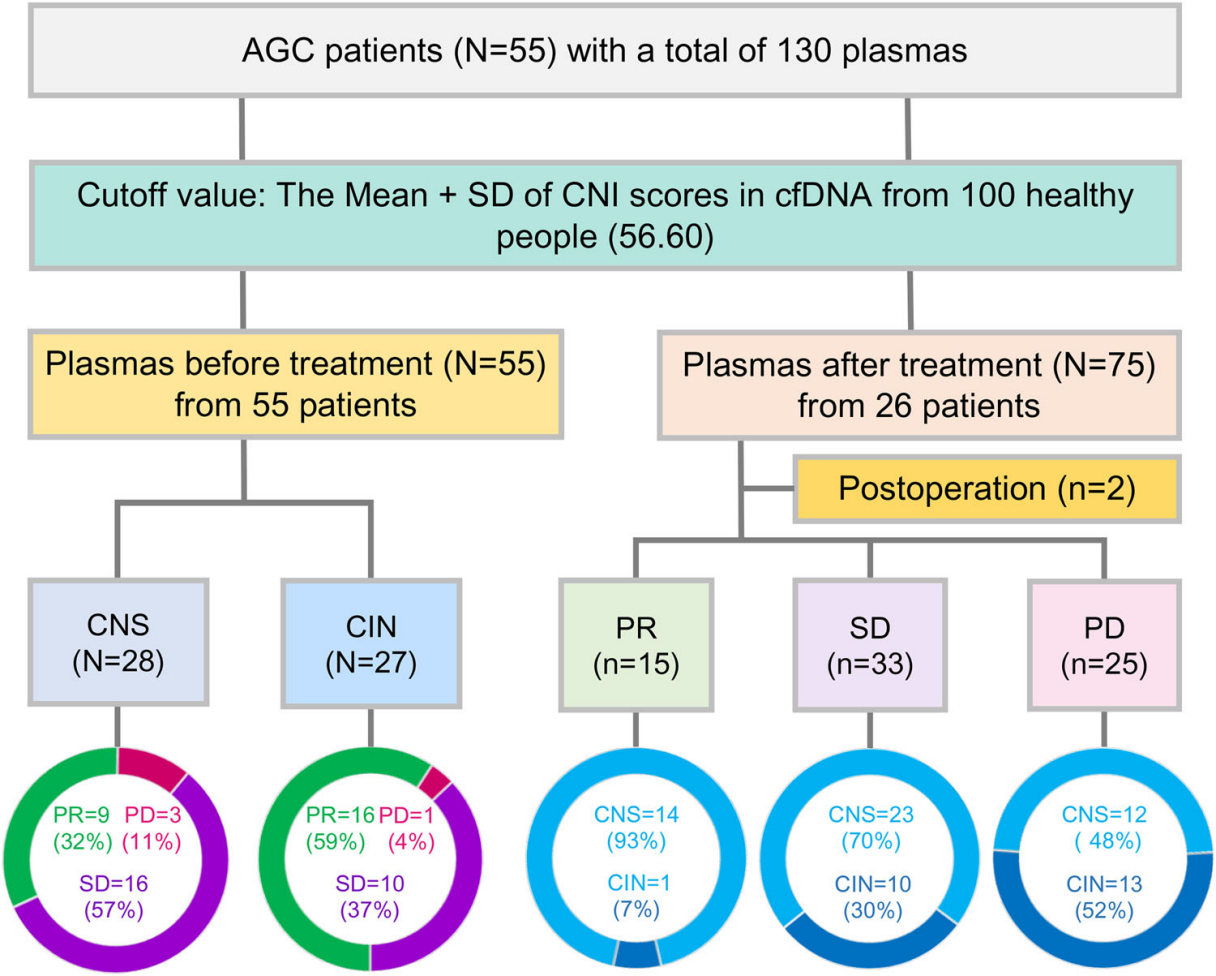
Schematic of 130 plasma samples and therapeutic response from 55 patients. For patients with pretherapeutic chromosomal instability, the response rate (59%, 16/27) was significantly higher than that in patients with chromosomal stability (32%, 9/28) (*P* = 0.043). The cutoff value of chromosomal instability was defined as the mean + SD of the CNI scores (56.60) in cfDNA from 100 healthy people. CNI scores also fluctuated during drug treatment among 26 patients with dynamic plasmas. CNI scores copy number instability score, CIN chromosomal instability, CNS chromosomal stability, PR partial response, SD stable disease, PD progressive disease

Among patients with chromosomal instability prior to treatment (*n* = 27), 16 patients (59%) achieved PR after drug treatment, 10 patients (37%) achieved SD, and 1 patients (4%) achieved progressive disease (PD). For patients with chromosomal stability (*n* = 28) prior to treatment, 9 patients (32%) achieved PR after treatment, 16 patients (57%) achieved SD, and 3 patients (11%) achieved PD. The response rate (59%, 16/27) of patients with chromosomal instability was higher than that (32%, 9/28) of patients with chromosomal stability (*P* = 0.043) (Fig. 3 and Table S3).

### Dynamic changes in CNI scores of ctDNA could indicate disease progression

**Fig. 4:**
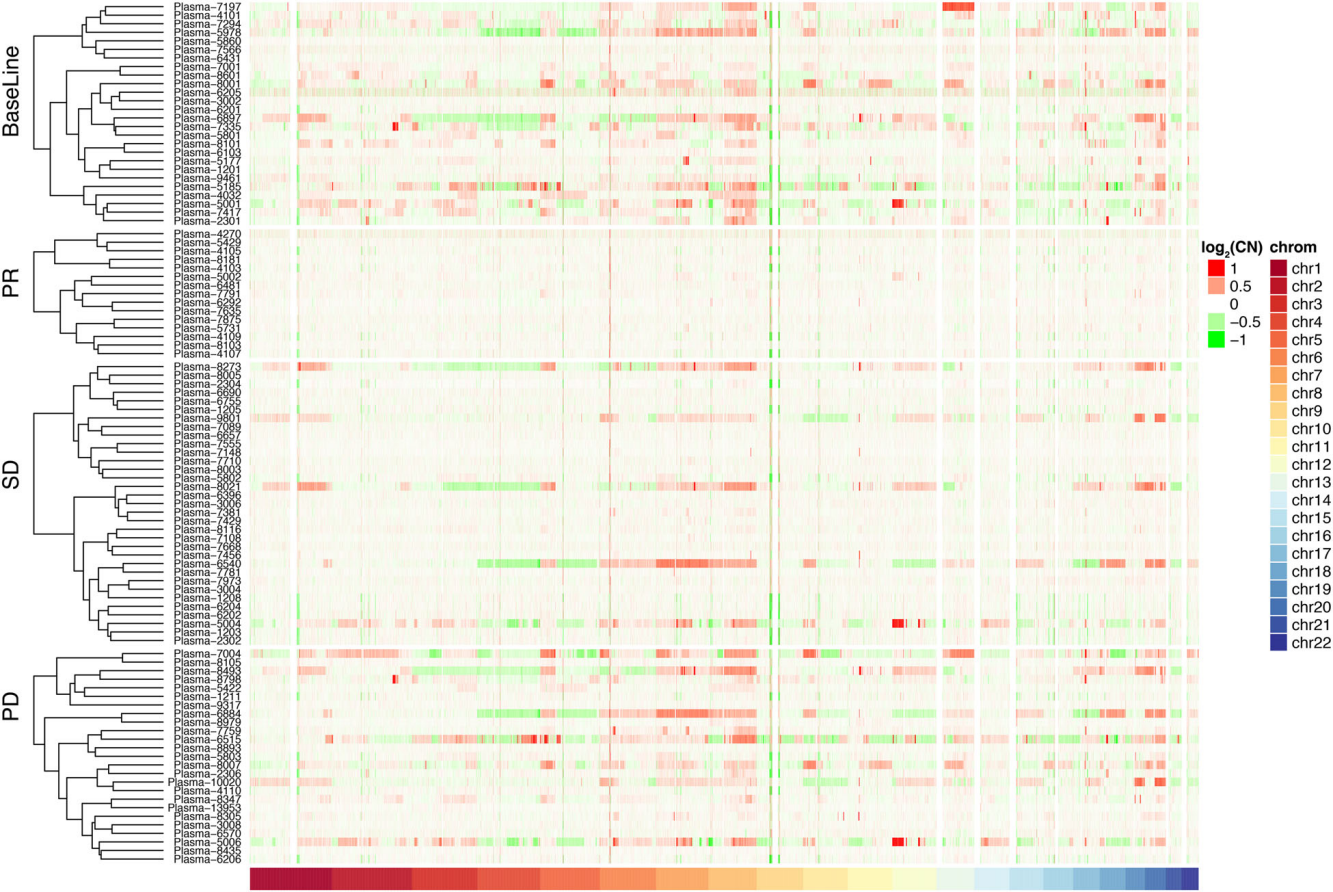
Cluster analysis of SCNV patterns and therapeutic response among 26 patients. In the dynamic administration of treatment, the CNI scores showed a sharp decline at PR, then achieved slow recovery at SD, and finally increased significantly at PD. The *x*-axis and *y*-axis represent the chromosomes’ loci and corresponding normalized copy numbers in plasma samples. PR partial response, SD stable disease, PD progressive disease, CN normalized copy number

**Fig. 5:**
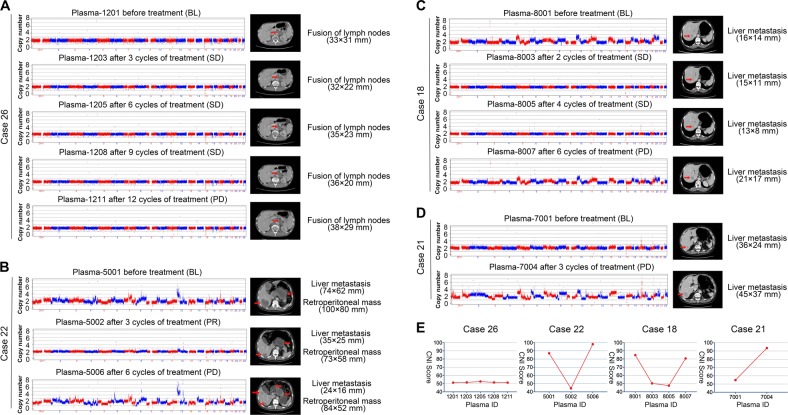
Dynamic changes in CNI scores of ctDNA and representative scan images. **a**–**d** Visualization of dynamic changes in CNI scores and representative scan images are presented for four patients (cases 26, 22, 18, and 21). PR partial response, SD stable disease, PD progressive disease. **e** The dynamic changes in CNI scores of ctDNA from four patients. CNI scores copy number instability score

Based on the clustering heatmap (Fig. 4), the CNI scores changed dynamically during therapy, which decreasing at PR or SD and re-increasing at PD. The detailed changes of CNI scores and therapeutic responses in 26 patients are shown in Figs. S2, S3, and Table S4. Four exemplary patients are also further demonstrated in Fig. 5. For case 26, with chromosomal stability (score, 51.23) at baseline, achieved SD after treatment with gradually swelling retroperitoneal lymph nodes until the progressive of disease (Fig. 5a). For case 22 (score, 84.76) and case 18 (score, 87.10), which had high levels of chromosomal instability at baseline, distant metastases were significantly shrunk with decreased CNI scores, and when patients developed resistance to treatment, the scores increased again (Fig. 5b, c). For case 21, with chromosomal stability (score, 54) at baseline, liver metastases increased rapidly after treatment, and the CNI score increased significantly (Fig. 5d). Changes in the CNI scores of these patients were observed during therapy (Fig. 5e).

**Fig. 6:**
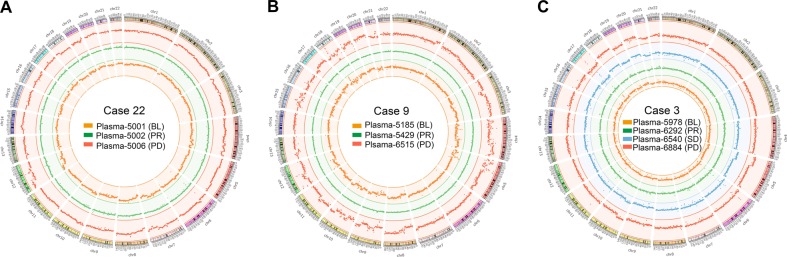
Dynamic SCNV patterns of ctDNA in three patients with acquired resistance. **a**–**c** The SCNV patterns at baseline, PR, SD, and PD in cases 22, 9, and 3, respectively. Orange, SCNV patterns at baseline (BL); green, SCNV patterns at partial response (PR); blue, SCNV patterns at stable disease (SD); red, SCNV patterns at progressive disease (PD). The Circos software package was used to plot genomic SCNVs

It seemed that the SCNA patterns of ctDNA with developed resistance were similar to those of paired pre-therapeutic ctDNA (Fig. 5). A circular map verified the identity of the SCNA pattern between pretherapeutic and resistant ctDNA, but the CNI score at resistance was lower than the pre-therapeutic level. Three representative patients are shown in Fig. 6.

## Discussion

Chromosomal instability has been reported to underpin intratumoral heterogeneity, accelerate clone evolution, drive phenotypic adaptation, finally resulting in a poor clinical outcome, and accelerating therapeutic resistance in various cancers^11,18,19^. Compared with single-nucleotide polymorphism, the analysis of the somatic copy number alteration (SCNA) pattern is a more universal approach based on chromosomal instability^13,15^. With the next-generation sequencing of ctDNA applied to the quantitative analysis of the SCNA pattern, it provides a sufficient method for early diagnosis of cancer^15,20^, prediction of treatment response^14^, and dynamic monitoring of acquired resistance^19^.

GC is featured by frequent SCNA and high-chromosomal instability. Among 295 patients with primary gastric adenocarcinomas from The Cancer Genome Atlas (TCGA) cohort, 50% showed chromosomal instability^4^. In the present study, chromosomal instability detected by whole ctDNA-based genome sequencing was observed in 27 of 55 patients (49%) prior to drug treatment. No significant relationship was found between patients’ characteristics and CNI scores, and we also did not find differences between survival and CNI scores (data not shown).

Chromosomal instability has been demonstrated to predict therapeutic response to radiotherapy, chemotherapy, and immunotherapy^14,21,22^. Recently, researchers observed a prediction accuracy of 83% of the quantified chromosomal instability and the prediction of SD vs. PD prior to standard imaging analysis^14^. In our research, we found that pre-treatment chromosomal instability could predict a higher response rate (59% vs. 32%, *P* *=* 0.043). Compared with baseline, the scores of 19 patients (73%) decreased after drug treatment, and greatly increased when disease progressed, to a level slightly lower than the baseline value. In addition, we also presented the dynamic changes of CEA, CA125, CA19-9, and CA72-4 of 14 patients throughout the treatment with series information of serum biomarkers (Fig. S4). The CNI scores decreased in 9/13 patients at PR or SD, accompanied by reduction levels of CEA, CA19-9, CA125, or CA72.4. These results derived from our relatively small sample are worth validating in future studies.

The noninvasiveness of ctDNA has been used to detect the acquired resistance mutations selected by treatment of nonsmall cell lung cancer, melanoma, and metastatic HER2-positive GC^7,23–25^. In the dynamic process of clone evolution under selective pressures induced by treatment, our research showed that, as expected, the CNI scores declined at PR and distinctly increased at PD. Compared with the traditional biomarkers, the CNI score elevated in 10/14 patients at progressive disease, accompanied by increasing levels of CEA, CA19-9, CA125, and CA72.4, alone or in combination (Fig. S4). More importantly, for case 22 with pre-therapeutic chromosomal instability, although the tumor biomarkers were decreased at stable disease (evaluated by RECIST criteria) after three cycles of treatment, we observed sharp increase of CNI score (44.28–91.41). This suggested that ctDNA-based CNI scores could serve as an early indicator of progression disease, whose power was comparable to the combination of CEA, CA19-9, CA125, and CA72-4.

Our previous research revealed a high concordance of *HER2* amplification between ctDNA and tumor tissues in 56 patients with AGC^16^. Furthermore, patients exhibited a decrease in the *HER2* copy number when they benefited from trastuzumab therapy, and achieved an increase at progressive disease. In the present study, among 17 patients with HER2-positive AGC who received anti-HER2 therapy, 13 (76%) achieved increases of the CNI scores, while 11 (65%) achieved increases of the *HER2* copy number, at progressive disease (Fig. S5). For the first time, we found that the efficacy of CNI score to monitor therapeutic response of anti-HER2 treatment was not inferior to the changes of *HER2* copy number.

The concept that spatiotemporal evolution of genomic clones is involved in drug resistance had been explored by several studies^25,26^. Multiregional and ctDNA-based next generation sequencing for intratumoral heterogeneity identification and genomic subclones detection could identify the dynamic emergence of resistant subclones during therapy^25,27^. Recently, the presence and size estimation of ibrutinib-resistant subclones at baseline in patients with chronic lymphocytic leukemia was demonstrated by droplet-based microfluidic technology and growth kinetic analyses^26^. In the present study, we found that the SCNA patterns of ctDNAs with acquired drug resistance were largely unchanged compared with baseline SCNV patterns, which suggested that multiple factors from different levels are involved in drug resistance.

## Conclusions

We employed a low-coverage WGS to quantify the chromosomal instability of plasma ctDNA and revealed the dynamic changes induced by drug treatment in AGC. Our finding suggests that chromosomal instability of ctDNA could be used to predict and monitor therapeutic response in GC, although validation in a larger cohort will be necessary.

## Materials and methods

### Study design

A cohort of 55 patients with histopathologically confirmed AGC who received chemotherapy or targeted therapy at Peking University Cancer Hospital were included in this study. Totally, 100 plasma samples from 100 healthy people, 55 pretherapeutic plasmas (26 has paired blood cells) from 55 AGC patients and 75 dynamic plasmas from 26 AGC patients were collected. The clinical data of patients was obtained from their medical records and the clinical response after drug treatment was evaluated by computed tomography and categorized as complete response, PR, SD, or PD, according to the RECIST 1.1 criteria^9^. This study was approved by the Medical Ethics Committee of Peking University Cancer Hospital, and written informed consent was obtained from all of the patients for their samples to be used in the future.

### Plasma collection and ctDNA extraction

Whole blood from patients was collected in cell-free DNA BCT tubes (Streck Laboratories, USA), and then centrifuged at 1600*g* for 10 min at 4 °C to separate plasma from blood cells. The supernatant was transferred into a fresh tube and centrifuged at 16,000*g* for another 10 min at 4 °C. ctDNA was extracted from a 1000 μL aliquot of plasma using a QIAamp Circulating Nucleic Acid Kit (Qiagen, Germany), and genomic DNAs from peripheral blood cells were extracted using the RelaxGene Blood DNA System (Tiangen Biotech Co., Ltd., China). The quality of DNA was examined by quantitative polymerase chain reaction and the 2100 Bioanalyzer (Agilent, USA). All of the samples were stored at −80 °C for further use.

### Whole-genome sequencing

Low-coverage whole-genome sequencing (LC WGS) based on ctDNA samples was performed to analyze SCNAs. More than 5 ng of the ctDNA was used to build the library, and the sequencing was performed on an Illumina HiSeq 2500 sequencer (Illumina, San Diego, CA, USA). Each sample had about 5 million paired-end reads, and the average length of each read was 100 bp. Over 91.92% of bases had a sequencing quality score ≥ Q30 in LC WGS. The QC of sequence data was performed as described previously^16^. The adapters and low-quality bases were filtered and trimmed from the raw data using Trimmomatic (version 0.35). High-quality reads were mapped to the reference genome (hg19) using BWA (version 0.7.12-r1039).

### Copy number analysis of ctDNA and CNI score calculation

Unique mapped reads were extracted from the alignment reads (BAM file). The whole reference genome was divided into non-overlapped observation windows (bins) with a size of 1000 kB. The read number and guanine-cytosine (GC) content were calculated in each bin. The bin read count was normalized based on the GC content and on a reference dataset to represent the relative copy number, which was reported accordingly^16^. we used R (version 3.0.0) to graph the relative copy number of each bin to visualize CNVs. The relative read number (RRN) of each bin was then segmented by circular binary segmentation (CBS) algorithms to merge bins with similar trends and calculate the final copy number segments. Then, we calculated the Z value of each bin according to the formula

$${z}_{\mathrm{i}} = \sqrt {\left| {{\it{log}}_2\left( {\frac{{{\it{x}}_i}}{2}} \right)} \right|},$$

where *x*_*i*_ is the relative copy number of each bin.

The CNI score was calculated according to the formula, as descripted previously^28^

$$\mathrm{CNI}\,\mathrm{score} = \mathop {\sum}\limits_{i = m_b}^{P_b} {\left| {Z_i} \right|},$$

where *m*_*b*_ and *p*_*b*_ are the bins ranked *m*% and *p*%, respectively, according to the *Z* value (*m* = 95, *p* = 99).

ChromGo (Yikon Genomics Inc., Shanghai, China) software was used to automatically analyze sequencing data and report abnormalities of chromosomes. We used software package Circos (http://circos.ca/), ideal for visualizing genome DNA, to plot patients’ SCNV at the baseline, PR, SD, and PD stages. For the clustering heatmap, we used the R package dendextend (https://cran.r-project.org/web/packages/dendextend/vignettes/introduction.html) for hierarchical clustering and the R package ComplexHeatmap (https://www.rdocumentation.org/packages/) to obtain the heatmap.

### Statistical analysis

A chi-square test was conducted to investigate the association of chromosomal instability with clinicopathological characteristics and the response rate of treatment. The significance of CNI scores between ctDNA of plasma and blood cells, as well as the differences before and after treatment, were determined with *t* test. The significance of CNI scores variance among ctDNA obtained from samples at baseline, PR, SD, and PD was measured by one-way ANOVA. Kaplan–Meier survival analysis was performed to compare the survival outcomes of patients with different CNI scores. *P* < 0.05 was considered statistically significant. Analyses were performed with SPSS 22.0 or GraphPad Prism 7.0.

## Supplementary information

Table S1

Table S2

Table S3

Table S4

Figure S1

Figure S2

Figure S3

Figure S4

Figure S5
