# Supplementary material for: Chromosomal instability of circulating tumor DNA reflect therapeutic responses in advanced gastric cancer
Source: Cell Death Dis. 2019 Sep 20;10(10):697. doi: 10.1038/s41419-019-1907-4 (PMC6754425; doi:10.1038/s41419-019-1907-4)
Supplement: Supplementary file 1 — Table S1 [file 41419_2019_1907_MOESM1_ESM.doc]

**Table S1. The detailed clinicopathological characteristics of 55** patients

| **Patient** | **Age** | **Gender** | **Tumor**  **location** | **Tumor differentiation** | **Lauren**  **classification** | **HER2**  **status** | **PFS**  **(month)** | **Treatment** | **Treatment**  **line** |
| --- | --- | --- | --- | --- | --- | --- | --- | --- | --- |
| 1 | 64 | Male | EGJ | Low | Intestinal | Positive | 7.1 | XP+Trastuzumab | First |
| 2 | 49 | Male | Non-EGJ | High | Intestinal | Positive | 8.0 | XELOX+Trastuzumab | First |
| 3 | 64 | Female | Non-EGJ | Low | Diffuse | Positive | 5.6 | XELOX+Trastuzumab | First |
| 4 | 46 | Male | Non-EGJ | Low | Diffuse | Positive | 7.5 | XELOX+Trastuzumab | First |
| 5 | 56 | Male | Non-EGJ | Middle | Intestinal | Positive | 8.1 | XELOX+Trastuzumab | First |
| 6 | 57 | Male | Non-EGJ | Low | Diffuse | Negative | 8.5 | Paclitaxel+Capecitabine | First |
| 7 | 42 | Male | Non-EGJ | Low | Intestinal | Negative | 9.7 | Paclitaxel+Capecitabine | First |
| 8 | 58 | Male | Non-EGJ | Middle | Mixed | Negative | 16.7 | Paclitaxel+Capecitabine | First |
| 9 | 29 | Male | EGJ | Middle | Intestinal | Negative | 7.2 | XELOX+Trastuzumab | First |
| 10 | 63 | Female | EGJ | Low | Diffuse | Positive | 4.5 | XP+Trastuzumab | First |
| 11 | 46 | Female | Non-EGJ | Low | Mixed | Positive | 9.1 | XELOX+Trastuzumab | First |
| 12 | 74 | Male | EGJ | Middle | Intestinal | Positive | 3.0 | XP+Trastuzumab+Pertuzumab/Placebo | First |
| 13 | 62 | Male | EGJ | Middle | Intestinal | Negative | 7.8 | XELOX | First |
| 14 | 46 | Male | Non-EGJ | Low | Diffuse | Negative | 43.6 | SOX | First |
| 15 | 63 | Male | EGJ | Low | Intestinal | Negative | 9.7 | XP | First |
| 16 | 56 | Female | Non-EGJ | Low | Intestinal | Negative | 2.6 | SOX | First |
| 17 | 57 | Male | Non-EGJ | High | Intestinal | Positive | 2.6 | Pyrotinib | Fourth |
| 18 | 62 | Female | Non-EGJ | Middle | Intestinal | Positive | 4.4 | Pyrotinib | Third |
| 19 | 52 | Male | EGJ | Middle | Intestinal | Positive | 1.5 | RC48 | Second |
| 20 | 49 | Male | Non-EGJ | Low | Mixed | Positive | 5.8 | Pyrotinib | Second |
| 21 | 65 | Male | EGJ | Middle | Intestinal | Positive | 2.0 | Pyrotinib | Second |
| 22 | 53 | Male | Non-EGJ | Low | Intestinal | Positive | 4.8 | XP+Trastuzumab+Pertuzumab/Placebo | First |
| 23 | 66 | Male | EGJ | Middle | Intestinal | Positive | 4.2 | Pyrotinib | Second |
| 24 | 59 | Male | EGJ | Low | Mixed | Positive | 3.8 | Pyrotinib | Third |
| 25 | 56 | Male | Non-EGJ | Low | Intestinal | Negative | 4.7 | Paclitaxel+Fruquintinib | Second |
| 26 | 56 | Male | Non-EGJ | Low | Diffuse | Positive | 5.8 | RC48 | First |
| 27 | 70 | Male | EJG | Middle | Intestinal | Positive | 8.8 | XP+Trastuzumab | First |
| 28 | 67 | Male | EJG | Middle | Intestinal | Positive | 5.6 | XP+Trastuzumab | First |
| 29 | 35 | Female | EJG | Low | Intestinal | Negative | 9.6 | Paclitaxel+Capecitabine | First |
| 30 | 64 | Female | Non-EJG | Middle | Intestinal | Negative | 9.6 | SOX | First |
| 31 | 70 | Male | Non-EJG | Low | Intestinal | Negative | 7.9 | XP | First |
| 32 | 50 | Male | Non-EJG | Middle | Mixed | Positive | 8.1 | XELOX | First |
| 33 | 29 | Female | EJG | Low | Mixed | Negative | 4.4 | Paclitaxel+Capecitabine | First |
| 34 | 44 | Male | EJG | Middle | Intestinal | Negative | 3.3 | XP | First |
| 35 | 67 | Male | Non-EJG | Middle | Diffuse | Negative | 8.6 | SOX | First |
| 36 | 51 | Female | Non-EJG | Low | Diffuse | Negative | 4.5 | Paclitaxel+Capecitabine | First |
| 37 | 53 | Male | Non-EJG | Middle | Intestinal | Positive | 9.6 | XELOX+Trastuzumab | First |
| 38 | 65 | Female | Non-EJG | Low | Diffuse | Negative | 3.7 | Paclitaxel+Capecitabine | First |
| 39 | 64 | Male | EJG | Low | Mixed | Negative | 3.9 | Paclitaxel+Capecitabine | First |
| 40 | 66 | Male | EJG | High | Intestinal | Negative | 7.8 | DOX | First |
| 41 | 43 | Male | Non-EJG | Middle | Intestinal | Negative | 1.4 | DCF | First |
| 42 | 72 | Male | EJG | Low | Intestinal | Negative | 9.9 | SOX | First |
| 43 | 56 | Male | Non-EJG | Low | Intestinal | Positive | 21.2 | XP+Trastuzumab+Pertuzumab/Placebo | First |
| 44 | 59 | Male | Non-EJG | Middle | Intestinal | Positive | 7.9 | XP+Trastuzumab+Pertuzumab/Placebo | First |
| 45 | 67 | Male | Non-EJG | High | Intestinal | Positive | 14.9 | XP+Trastuzumab+Pertuzumab/Placebo | First |
| 46 | 46 | Female | EJG | Low | Intestinal | Positive | 10.9 | XELOX+Trastuzumab | First |
| 47 | 59 | Male | Non-EJG | Middle | Intestinal | Positive | 7.1 | XELOX+Trastuzumab | First |
| 48 | 60 | Male | Non-EJG | Middle | Intestinal | Positive | 21.2 | XP+Trastuzumab+Pertuzumab/Placebo | First |
| 49 | 74 | Male | EJG | Middle | Intestinal | Positive | 19.2 | XP+Trastuzumab+Pertuzumab/Placebo | First |
| 50 | 62 | Male | Non-EJG | Low | Intestinal | Negative | 3.0 | Paclitaxel+Capecitabine | First |
| 51 | 65 | Male | Non-EJG | Middle | Intestinal | Positive | 10.5 | XP+Trastuzumab+Pertuzumab/Placebo | First |
| 52 | 80 | Male | EJG | Middle | Intestinal | Negative | 6.5 | XELOX | First |
| 53 | 29 | Female | Non-EJG | Low | Diffuse | Positive | 2.9 | XELOX+Trastuzumab | Second |
| 54 | 54 | Male | Non-EJG | Low | Intestinal | Negative | 2.9 | Paclitaxel +S-1 | First |
| 55 | 58 | Male | Non-EJG | Low | Intestinal | Negative | 8.1 | Paclitaxel +Oxaliplatin+S1 | First |
