# Supplementary material for: Chromosomal instability of circulating tumor DNA reflect therapeutic responses in advanced gastric cancer
Source: Cell Death Dis. 2019 Sep 20;10(10):697. doi: 10.1038/s41419-019-1907-4 (PMC6754425; doi:10.1038/s41419-019-1907-4)
Supplement: Supplementary file 2 — Table S2 [file 41419_2019_1907_MOESM2_ESM.doc]

**Table S2. The copy number instability (CNI) score of cfDNA from plasmas in 100 healthy people.**

| **ID** | **CNI Score** | **ID** | **CNI Score** |
| --- | --- | --- | --- |
| 1 | 49.55 | 51 | 55.28 |
| 2 | 50.59 | 52 | 47.81 |
| 3 | 52.68 | 53 | 50.55 |
| 4 | 57.66 | 54 | 48.97 |
| 5 | 54.21 | 55 | 48.80 |
| 6 | 57.09 | 56 | 47.43 |
| 7 | 53.91 | 57 | 56.38 |
| 8 | 53.53 | 58 | 54.75 |
| 9 | 52.74 | 59 | 46.63 |
| 10 | 52.74 | 60 | 59.78 |
| 11 | 56.82 | 61 | 56.49 |
| 12 | 52.00 | 62 | 51.87 |
| 13 | 56.60 | 63 | 51.75 |
| 14 | 53.47 | 64 | 51.08 |
| 15 | 59.29 | 65 | 49.13 |
| 16 | 53.83 | 66 | 53.51 |
| 17 | 53.79 | 67 | 53.72 |
| 18 | 50.19 | 68 | 46.74 |
| 19 | 52.86 | 69 | 46.99 |
| 20 | 53.19 | 70 | 47.11 |
| 21 | 54.79 | 71 | 46.86 |
| 22 | 51.26 | 72 | 46.08 |
| 23 | 53.29 | 73 | 53.46 |
| 24 | 58.85 | 74 | 51.61 |
| 25 | 53.44 | 75 | 52.60 |
| 26 | 56.38 | 76 | 54.35 |
| 27 | 53.33 | 77 | 53.60 |
| 28 | 56.78 | 78 | 57.10 |
| 29 | 56.66 | 79 | 53.62 |
| 30 | 53.90 | 80 | 54.25 |
| 31 | 57.88 | 81 | 61.21 |
| 32 | 56.74 | 82 | 54.78 |
| 33 | 54.59 | 83 | 60.78 |
| 34 | 54.98 | 84 | 54.61 |
| 35 | 52.14 | 85 | 60.55 |
| 36 | 54.96 | 86 | 55.99 |
| 37 | 53.00 | 87 | 49.87 |
| 38 | 48.81 | 88 | 49.76 |
| 39 | 51.85 | 89 | 50.62 |
| 40 | 52.03 | 90 | 55.47 |
| 41 | 52.34 | 91 | 58.41 |
| 42 | 51.92 | 92 | 52.79 |
| 43 | 53.15 | 93 | 61.27 |
| 44 | 52.24 | 94 | 50.69 |
| 45 | 49.68 | 95 | 55.56 |
| 46 | 53.15 | 96 | 52.00 |
| 47 | 52.48 | 97 | 50.16 |
| 48 | 52.74 | 98 | 52.57 |
| 49 | 53.88 | 99 | 54.97 |
| 50 | 50.87 | 100 | 49.71 |
