# Supplementary material for: Chromosomal instability of circulating tumor DNA reflect therapeutic responses in advanced gastric cancer
Source: Cell Death Dis. 2019 Sep 20;10(10):697. doi: 10.1038/s41419-019-1907-4 (PMC6754425; doi:10.1038/s41419-019-1907-4)
Supplement: Supplementary file 3 — Table S3 [file 41419_2019_1907_MOESM3_ESM.doc]

**Table S3. The copy number instability (CNI) score of ctDNA from baseline plasmas and corresponding therapeutic response**

| **Patient** | **Plasma** | **CNI Score** | **chromosomal instability** | **Therapeutic response** |
| --- | --- | --- | --- | --- |
| 1 | 7566 | 49.41 | Stable | PR |
| 2 | 6205 | 64.36 | Instable | PR |
| 3 | 5978 | 97.43 | Instable | PR |
| 4 | 5177 | 63.93 | Instable | PR |
| 5 | 7335 | 86.05 | Instable | SD |
| 6 | 6897 | 90.47 | Instable | SD |
| 7 | 6103 | 44.69 | Stable | SD |
| 8 | 5860 | 48.69 | Stable | SD |
| 9 | 5185 | 94.43 | Instable | PR |
| 10 | 7417 | 64.49 | Instable | PR |
| 11 | 4032 | 59.49 | Instable | PR |
| 12 | 9461 | 75.42 | Instable | SD |
| 13 | 7197 | 91.65 | Instable | PR |
| 14 | 7294 | 70.11 | Instable | PR |
| 15 | 6431 | 53.47 | Stable | SD |
| 16 | 8601 | 64.35 | Stable | PD |
| 17 | 8101 | 68.66 | Instable | PR |
| 18 | 8001 | 84.76 | Instable | SD |
| 19 | 5801 | 61.67 | Instable | SD |
| 20 | 4101 | 62.65 | Instable | PR |
| 21 | 7001 | 54.79 | Stable | PD |
| 22 | 5001 | 87.10 | Instable | PR |
| 23 | 3001 | 48.96 | Stable | SD |
| 24 | 6201 | 52.25 | Stable | SD |
| 25 | 2301 | 67.67 | Instable | SD |
| 26 | 1201 | 51.23 | Stable | SD |
| 27 | 7213 | 56.81 | Stable | PR |
| 28 | 6768 | 49.06 | Stable | PR |
| 29 | 6715 | 50.50 | Stable | SD |
| 30 | 6420 | 52.65 | Stable | SD |
| 31 | 5231 | 51.19 | Stable | SD |
| 32 | 6738 | 52.32 | Stable | PR |
| 33 | 5029 | 91.75 | Instable | SD |
| 34 | 5142 | 48.92 | Stable | SD |
| 35 | 4276 | 52.24 | Stable | PR |
| 36 | 7711 | 49.52 | Stable | SD |
| 37 | 7277 | 61.98 | Instable | SD |
| 38 | 6320 | 55.54 | Stable | PR |
| 39 | 6301 | 50.43 | Stable | SD |
| 40 | 7118 | 51.84 | Stable | PD |
| 41 | 6554 | 61.49 | Instable | PR |
| 42 | 6221 | 47.85 | Stable | SD |
| 43 | 1101 | 62.99 | Instable | PR |
| 44 | 6101 | 59.38 | Instable | SD |
| 45 | 3101 | 55.93 | Stable | PR |
| 46 | 3501 | 53.26 | Stable | PR |
| 47 | 6601 | 79.60 | Instable | PR |
| 48 | 2101 | 52.34 | Stable | PR |
| 49 | 9001 | 81.39 | Instable | PR |
| 50 | 1501 | 63.08 | Instable | SD |
| 51 | 9101 | 51.34 | Stable | PR |
| 52 | 5701 | 50.56 | Stable | PD |
| 53 | 5501 | 51.57 | Stable | SD |
| 54 | 7401 | 51.06 | Stable | SD |
| 55 | 6801 | 52.96 | Stable | SD |
