# Supplementary material for: Chromosomal instability of circulating tumor DNA reflect therapeutic responses in advanced gastric cancer
Source: Cell Death Dis. 2019 Sep 20;10(10):697. doi: 10.1038/s41419-019-1907-4 (PMC6754425; doi:10.1038/s41419-019-1907-4)
Supplement: Supplementary file 4 — Table S4 [file 41419_2019_1907_MOESM4_ESM.doc]

**Table S4. The copy number instability (CNI) score of plasma ctDNA**

**during the drug treatment**

| **Patient** | **Plasma** | **CNI Score** | **chromosomal instability** | **Time point** |
| --- | --- | --- | --- | --- |
| 1 | 7566 | 49.41 | Stable | Baseline |
|  | 7875 | 46.29 | Stable | PR |
|  | 8893 | 50.96 | Stable | PD |
| 2 | 6205 | 64.36 | Instable | Baseline |
|  | 6481 | 50.30 | Stable | PR |
|  | 7381 | 52.08 | Stable | SD |
|  | 7759 | 60.12 | Instable | PD |
| 3 | 5978 | 97.43 | Instable | Baseline |
|  | 6292 | 52.07 | Stable | PR |
|  | 6540 | 92.41 | Instable | SD |
|  | 6884 | 90.21 | Instable | PD |
| 4 | 5177 | 63.93 | Instable | Baseline |
|  | 5731 | 50.84 | Stable | PR |
|  | 6570 | 51.61 | Stable | PD |
| 5 | 7335 | 86.05 | Instable | Baseline |
|  | 7668 | 51.02 | Stable | SD |
|  | 7973 | 51.12 | Stable | SD |
|  | 8798 | 65.02 | Instable | PD |
| 6 | 6897 | 90.47 | Instable | Baseline |
|  | 7148 | 49.32 | Stable | SD |
|  | 7456 | 52.46 | Stable | SD |
|  | 8021 | 89.41 | Instable | SD |
|  | 8273 | 88.78 | Instable | SD |
|  | 8493 | 91.06 | Instable | PD |
| 7 | 6103 | 44.69 | Stable | Baseline |
|  | 6396 | 46.98 | Stable | SD |
|  | 6657 | 47.27 | Stable | SD |
|  | 7108 | 48.51 | Stable | SD |
|  | 7429 | 47.25 | Stable | SD |
|  | 7710 | 51.41 | Stable | SD |
|  | 9317 | 48.37 | Stable | PD |
| 8 | 5860 | 48.69 | Stable | Baseline |
|  | 6690 | 50.02 | Stable | SD |
|  | 8979 | 49.58 | Stable | PD |
| 9 | 5185 | 94.43 | Instable | Baseline |
|  | 5429 | 46.37 | Stable | PR |
|  | 6515 | 98.05 | Instable | PD |
| 10 | 7417 | 64.49 | Instable | Baseline |
|  | 7791 | 52.51 | Stable | PR |
|  | 8116 | 52.66 | Stable | SD |
|  | 8437 | 57.00 | Instable | PD |
| 11 | 4032 | 59.49 | Instable | Baseline |
|  | 4270 | 51.99 | Stable | PR |
|  | 5062 | 48.24 | Stable | Postoperative baseline |
|  | 5422 | 50.05 | Stable | PD |
| 12 | 9461 | 75.42 | Instable | Baseline |
|  | 9801 | 87.09 | Instable | SD |
|  | 10020 | 90.77 | Instable | PD |
| 13 | 7197 | 91.65 | Instable | Baseline |
|  | 7555 | 45.06 | Stable | SD |
|  | 8181 | 49.26 | Stable | PR |
|  | 8435 | 46.14 | Stable | PD |
| 14 | 7294 | 70.11 | Instable | Baseline |
|  | 7635 | 45.32 | Stable | PR |
|  | 8638 | 43.99 | Stable | Postoperative baseline |
| 15 | 6431 | 53.47 | Stable | Baseline |
|  | 6755 | 53.66 | Stable | SD |
|  | 7089 | 45.14 | Stable | SD |
|  | 7781 | 46.74 | Stable | SD |
|  | 8305 | 61.83 | Instable | PD |
| 16 | 8601 | 64.35 | Instable | Baseline |
|  | 13953 | 46.86 | Stable | PD |
| 17 | 8101 | 68.66 | Instable | Baseline |
|  | 8103 | 47.74 | Stable | PR |
|  | 8105 | 46.48 | Stable | PD |
| 18 | 8001 | 84.76 | Instable | Baseline |
|  | 8003 | 50.22 | Stable | SD |
|  | 8005 | 47.55 | Stable | SD |
|  | 8007 | 80.69 | Instable | PD |
| 19 | 5801 | 61.67 | Instable | Baseline |
|  | 5802 | 53.39 | Stable | SD |
|  | 5803 | 53.72 | Stable | PD |
| 20 | 4101 | 62.65 | Instable | Baseline |
|  | 4103 | 47.49 | Stable | PR |
|  | 4105 | 49.38 | Stable | PR |
|  | 4107 | 49.72 | Stable | PR |
|  | 4109 | 49.21 | Stable | PR |
|  | 4110 | 50.05 | Stable | PD |
| 21 | 7001 | 54.79 | Stable | Baseline |
|  | 7004 | 93.51 | Instable | PD |
| 22 | 5001 | 87.10 | Instable | Baseline |
|  | 5002 | 44.28 | Stable | PR |
|  | 5004 | 91.41 | Instable | SD |
|  | 5006 | 98.15 | Instable | PD |
| 23 | 3001 | 48.96 | Stable | Baseline |
|  | 3004 | 49.20 | Stable | SD |
|  | 3006 | 55.93 | Stable | SD |
|  | 3008 | 46.99 | Stable | PD |
| 24 | 6201 | 52.25 | Stable | Baseline |
|  | 6202 | 50.70 | Stable | SD |
|  | 6204 | 49.70 | Stable | SD |
|  | 6206 | 50.45 | Stable | PD |
| 25 | 2301 | 67.67 | Instable | Baseline |
|  | 2302 | 51.69 | Stable | SD |
|  | 2304 | 51.45 | Stable | SD |
|  | 2306 | 55.77 | Stable | PD |
| 26 | 1201 | 51.23 | Stable | Baseline |
|  | 1203 | 51.52 | Stable | SD |
|  | 1205 | 52.65 | Stable | SD |
|  | 1208 | 51.46 | Stable | SD |
|  | 1211 | 51.28 | Stable | PD |
