# Supplementary figures and images for: Chromosomal instability of circulating tumor DNA reflect therapeutic responses in advanced gastric cancer
Source: Cell Death Dis. 2019 Sep 20;10(10):697. doi: 10.1038/s41419-019-1907-4 (PMC6754425; doi:10.1038/s41419-019-1907-4)

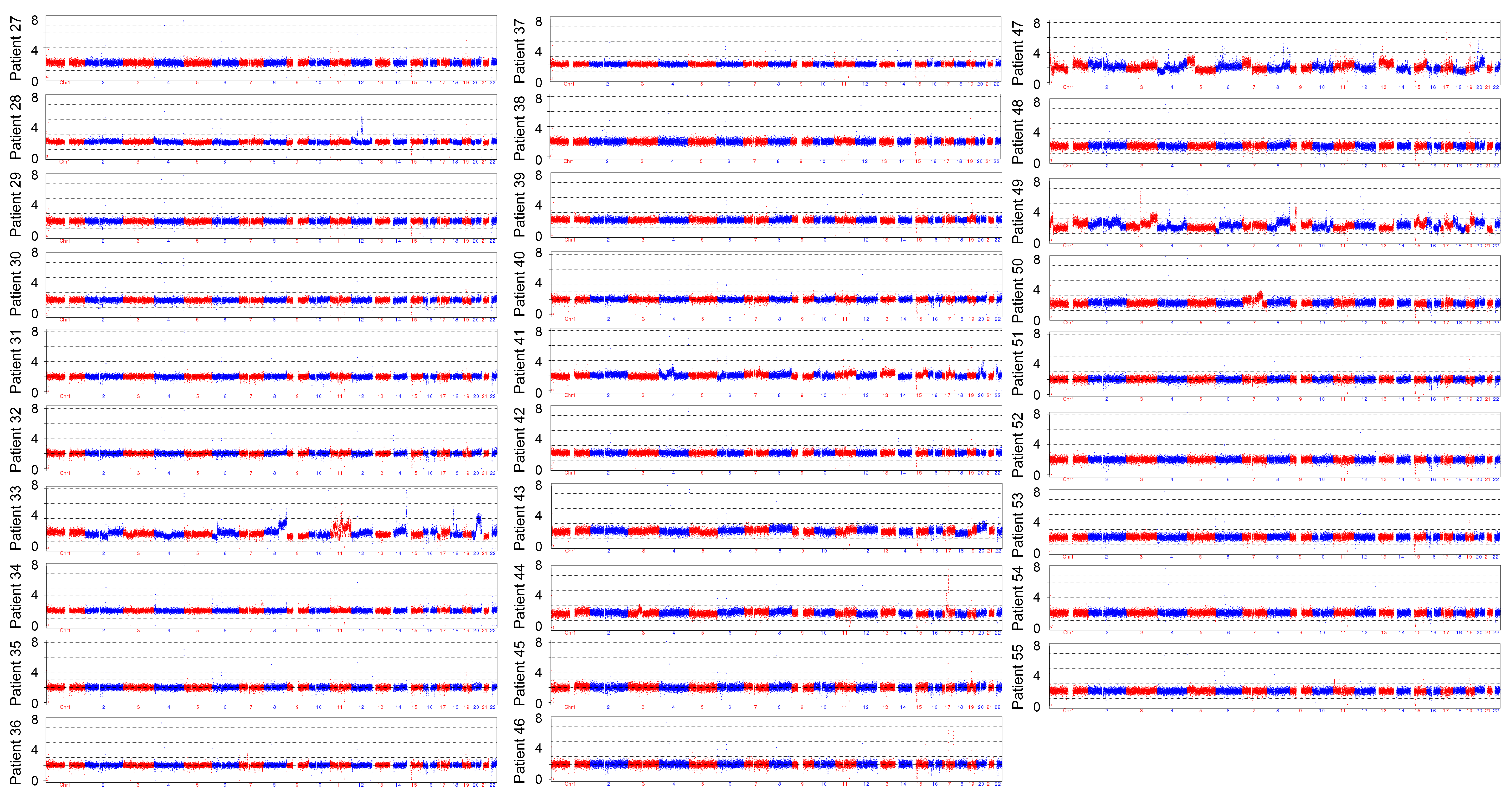

Supplement: Supplementary file 5 — Figure S1 [file 41419_2019_1907_MOESM5_ESM.png]

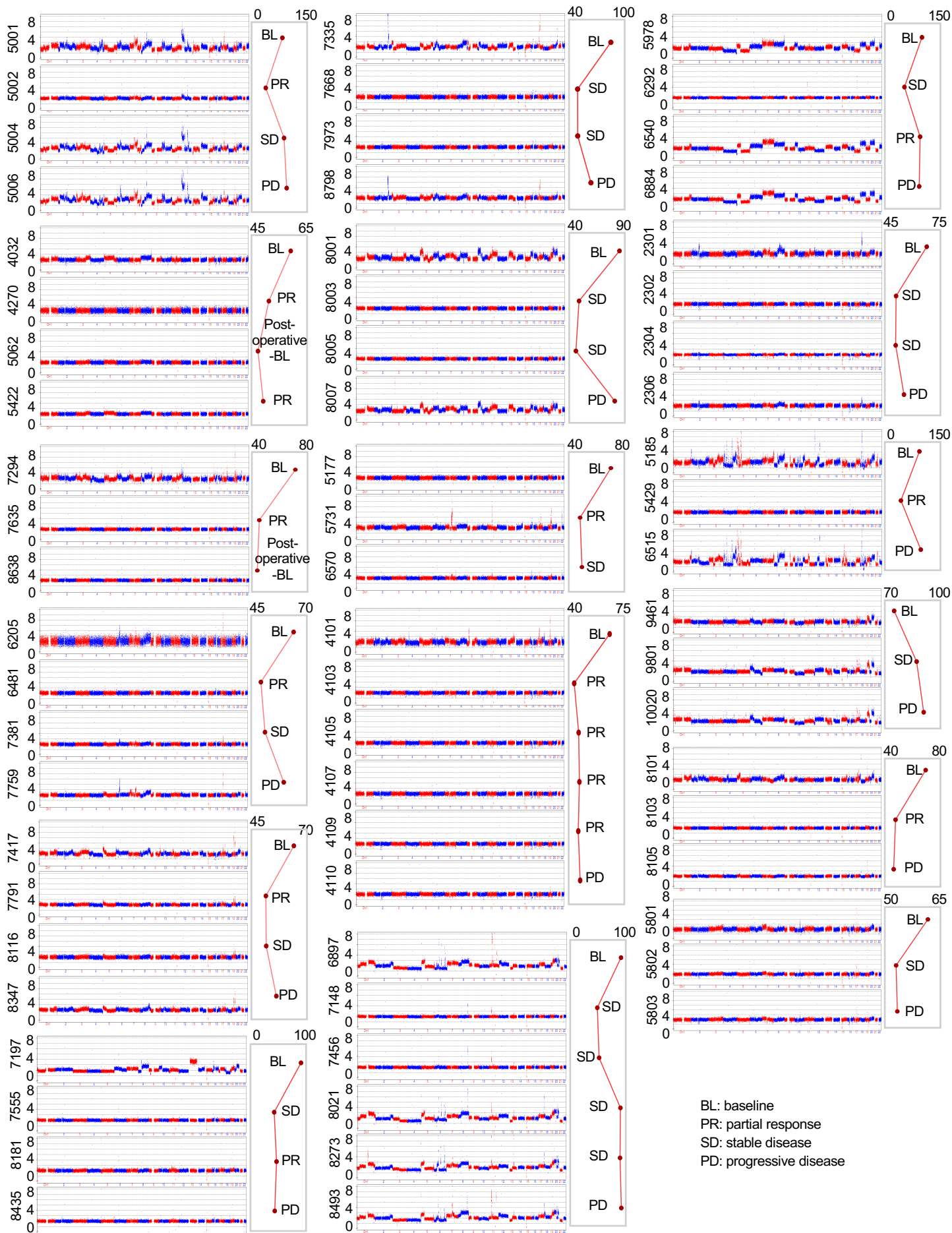

Supplement: Supplementary file 6 — Figure S2 [file 41419_2019_1907_MOESM6_ESM.pdf]

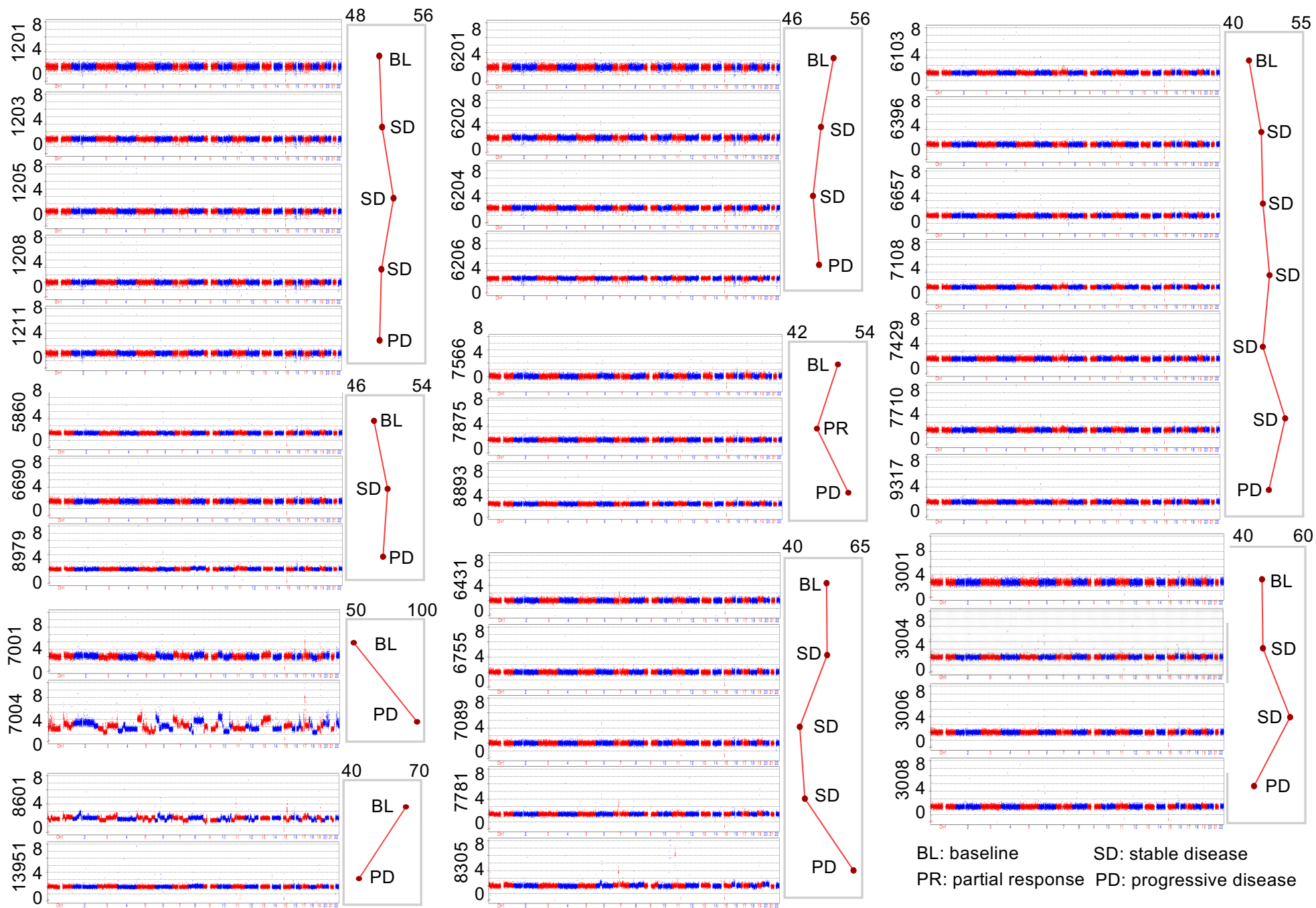

Supplement: Supplementary file 7 — Figure S3 [file 41419_2019_1907_MOESM7_ESM.pdf]
